# A Modern Treatment of Hemorrhoids

**Published:** 1895-08

**Authors:** T. J. Bennett

**Affiliations:** Austin, Texas


### A MODERN TREATMENT OF HEMERRHOIDS.

BY T. J. BENNETT, M. D., AUSTIN, TEXAS.

[Read before the Austin District Medical Society.]

"VARICOSITIES of the anal or rectal vessels" was once considered a good difinition of hemorrhoids. It is yet a good brief definition for certain kinds and certain stages of development of these tumors, but clinical observation and pathological research have demonstrated so many different characteristics that one would hardly recognize all the tumors in any given case of hemorrhoids by that test alone.

An extravasation of blood into the connective tissue, external to the sphincter, for instance, forming a tumor, is the initial step, it is true, in the formation of a pile, but long before the surgeon is called in, the tumor, under continuous inflammatory stimulus, may have lost its original features and become a true hypertrophy, a veritable "tab." There is a tendency to hypertrophy of all hemorrhoidal tumors, but less so to the internal than to the external variety. This change, together with other changes, which take place in the pathology of the tumors subsequent to their formation, are really to be found in nearly all cases in some degree, depending upon the length of time they have existed and upon the severity or suddenness of the attack. If the onslaught is sudden and the tumor is large, there is usually to be found a large collection of fresh blood, or a blood clot, in the center of the mass. On the other hand, if the attack is slow in its development, the tumor is filled with fluid blood which flows on, the mass being compressible. The walls of all these tumors may be thinner or thicker, as they are recent or old, or as they are less or greatly inflamed.

I need hardly say that the classification of hemorrhoids is the same old division into external and internal, "for convenience," and that the external variety is formed from the superficial or cutaneous vessels,—that is, from the inferior and middle hemorrhoidal veins, and are therefore connected with the general circulation; while the internal arise from the venules and arterioles of the middle and superior hemorrhoidal vessels, and are connected with the portal circulation. It is well always, however, to keep in mind these anatomical facts. The divisions and subdivisions of this classification are based upon the pathology and clinical features of the lesions. Dr. Tuttle, of the New York Polyclinic, makes as simple and as reasonable a subclassification as is warranted at this time. He divides external piles into four varieties, viz.: Thrombotic, varicose, inflammatory, and connective tissue piles; and of the internal variety he says there are practically but two kinds, the capillary, or nevoid, and the varicose. The pathological and physical characteristics of piles are so graphically suggested by the names used in this classification, that I deem a further description wholly unnecessary for your clear understanding and appreciation. With the clinical features of piles you are all abundantly familiar. Many of you, no doubt, could speak personally on the subject, the disease is so common.

From the foregoing, seeing that hemorrhoids exist in all shades of severity from the mere pin-head extravasation causing an uneasy sensation and no special annoyance, to the hen-egg size, attended with extreme pain and a high grade of inflammation, it is reasonable to expect as great a variety of treatment. But before entering upon the treatment of piles, let me emphasize the necessity of making strict inquiry into the causation. That far the majority of cases are caused directly by neglect of the bowels,—not going to stool at the proper time, being constipated, straining, etc., all will agree. But there are cases, and many of them too, produced by constitutional causes, such as congestion of the liver, various neoplasms which cause pressure on the return flow of blood, inflammation of the rectum, child birth, cystitis, and other conditions which produce continuous straining. These causes have to be taken into account in a rational treatment of the disease. It is also evident, from the nature and history of this disease, that all cases do not demand a surgical operation for their cure. In fact, there are many cases so slight, in the amount of inconvenience they occasion, that they do not call for any treatment at all, local or otherwise. There are many cases, moreover, in fact the majority, if not seventy-five per cent. of all cases, that are amenable to local or constitutional treatment, or both combined.

In general terms, hemorrhoids are amenable to local and constitutional treatment when the tumors are small and recent; when they are highly inflammatory, involving either the anal margin or higher up; when they are varicose and simple, and when they are not extremely painful nor bleeding. But hemorrhage is nearly always caused by fissures, and not by hemorrhoids pure and simple. On the other hand, when the tumors are of long standing, large and painful; when they are not compressible, and are ulcerated and are filled with clots; when they are complicated with fissures and hemorrhage; when the sphincter has become so strong by the long stimulus of the inflammation that it is difficult to evacuate the bowels, and more difficult to return the tumors and thickened mucous membrane which may be protruding, operative interference is indicated, and in nearly all such cases, imperative. It is fortunate, however, that nearly all of the operative procedures will succeed in curing the cases, if properly performed, and some little judgment used in the selection of cases and operation to be employed.

Local treatment consists of rest in bed, the application of cold water, or hot water, enemas of either, to suit the individual case, and the use of some such ointment as the following:

℞ Bismuth subgallate ..............grs. 60
Europhen .......................grs. 20
Vaseline .................... .....oz. 1

M. ft. ungt. Sig.: Apply well two or three times a day.

If the mucous membrane is very much irritated or inflamed and much pain is complained of, an anodyne, such as four or five grains of cocaine or morphine may be added to the ointment. Laxatives are to be given as they are indicated to keep the bowels open and the actions soft. For this purpose I am inclined to tablets of cream tartar, two and half grains, and sulphur four grains, each, administered two or three times daily. When the liver is congested, of course, depletives, such as hyposulphite of soda or phosphate of soda, may be given, and other treatment administered as indicated. And so other constitutional measures must be employed to meet other constitutional causes.

The standard operations for hemorrhoids,—at least, the two prominent operations, found in our standard books on the subject, are the method by clamp and cautery, and that by the ligature. There are more advocates of one or the other of these operations than any other that has ever been suggested. It can be said of them that they are successful when properly performed, but they have their disadvantages in not being as simple as some other methods are, and that they cause more pain, and frequently prolong the convalescence unduly. But these objections may not be regarded as serious, since the operations are practically safe and successful.

As a matter of personal choice and familiarity, I employ another operation which I hope to be able to show further on.

Of the more prominent operative procedures, other than the two mentioned above, are the methods by injection, crushing, snaring, as suggested by Kellogg, and Whitehead's dissection operation.

I think I can safely say that the injection plan is pretty generally condemned by the better class of medical men everywhere, as being unsafe, less frequently successful and tedious, in that it often requires many weeks to complete a cure.

The method by crushing is an old procedure, recently revived by Allingham, Jr., and comes nearest the operation which I shall speak of presently.

Whitehead's operation is certainly limited in its field of usefulness. The only kind of a case that I should employ this operation upon, would be one complicated with prolapsed rectum of long duration, and where the tumors are large and ulcerated and the connective tissue is much thickened by long continued inflammatory action.

Pratt's operation is said to be a modification of Whitehead's, but in just what the modification consists I am unable to state, unless it is in a more extensive use of the procedure. I have seen a case operated upon by Pratt, in which a huge failure was the result. The patient said he was not much inconvenienced before the operation, and submitted to it only because of the reputation around Chicago of the Pratt method. Like the epitaph on an Italian tombstone:

"I was well, I wished to be better,—
I sent for a doctor, took physic and died."

The operation consists in dissecting up the bowel, beginning at the anal margin, and drawing down and out the gut and cutting it off above the diseased portion, even including the normal pouches whose function is to retain and supply lubricant material with which to facillitate the escape of fecal matter. The bowel is then sewed to the margin of the true skin. The patient above referred to complained that his rectum was continuously dry, and gave him much pain on this account whenever his bowels acted. I don't think we can too strongly condemn this operation for general use. It is only singular that it should have ever received any recognition outside of the limitation above referred to.

The operation, to which I now call your attention, is one I have used for ten years, nearly exclusively, and which I am constrained to believe, is a model procedure.

It consists in all of the approved preliminary steps to any operation on the rectum, viz., thoroughly emptying the bowel by a purgative several hours before the operation, flushing with salt water just previous to commencing, rendering the rectum and anal surroundings as nearly aseptic as possible, divulsing the sphincter, etc. After all of this is satisfactorily attended to, the patient having been brought under an anæsthetic, the next step is grasping the tumors longitudinally with a forceps (Pean's pedicle forceps, which is slightly curved on the flat, answers every purpose) and breaking them down with the point of an ordinary bistoury so as to leave a roughened surface. If the surface is too ragged, and the fragments hang in too great length, the hanging pieces may be caught up and scraped off to suit. The surface being a torn one, permitting blood to rapidly coagulate upon it, there is no danger of hemorrhage, and the wound can not fail to get well early and perfectly. The procedure is not by crushing, but more by scraping, after once the mass has been broken down. It is immaterial how you break the tumor down. You may incise it, cutting it through and through in several places, and then scrape it down to the size you want it, which consists in destroying just the portion above the grasp of the forceps.

This operation, I believe, is adapted to any case demanding operative interference at all, except possibly a case in which the Whitehead is indicated.

The surfaces are in as good condition to heal as you could hope to leave them, and they do heal in less time than it takes for a ligature to slough off, and with very much less pain. In fact, there is usually no pain at all, because the strong pressure of the sphincter has been taken off, and if the patient is kept quiet, there is absolutely nothing to produce pain. The surfaces get well, too, earlier than if they had been cauterized, and without any risk of a contracting cicatrix remaining.

I consider that the divulsion of the sphincter has a great deal to do in relieving hemorrhoids. The smaller tumors get well without being operated upon, by thus removing the extra pressure from them.

I might say that indiscriminate divulsing of the sphincter is neither a safe thing to do, nor necessary in all cases. The best way to tell when it would be necessary to paralyze the sphincter, in any given case, is to try the strength of the muscle. If it is weak, as is sometimes the case in very debilitated persons, or in old people, or in women with lacerated perineums, there would be great danger of permanently disabling the sphincter for life; and besides, the divulsing is not indicated, as there is no pressure, and it should not be done at all.

The length of time usually required for a patient to remain in bed or his room is three to five days. The bowels may be emptied on the second day by an enema or a glass of bitter water. No pain. The sphincter muscle regains the necessary tention to prevent involuntary actions, in two or three days, and the patient is well.

---

**Old Prescriptions.**—One hundred and fifteen thousand prescriptions, being part of the stock of a druggist, were recently sold by the sheriff for $2300. If druggists were not permitted to refill prescriptions this stock would not be worth as much as was paid for it.—*Medical Examiner.*

